# The consciousness state space (CSS)—a unifying model for consciousness and self

**DOI:** 10.3389/fpsyg.2014.00341

**Published:** 2014-04-29

**Authors:** Aviva Berkovich-Ohana, Joseph Glicksohn

**Affiliations:** ^1^Department of Neurobiology, Weizmann Institute of ScienceRehovot, Israel; ^2^Department of Criminology, Bar-Ilan UniversityRamat Gan, Israel; ^3^The Leslie and Susan Gonda (Goldschmied) Multidisciplinary Brain Research Center, Bar-Ilan UniversityRamat Gan, Israel

**Keywords:** consciousness, time, awareness, emotion, self, default mode network, flow experience, meditation

## Abstract

Every experience, those we are aware of and those we are not, is embedded in a subjective timeline, is tinged with emotion, and inevitably evokes a certain sense of self. Here, we present a phenomenological model for consciousness and selfhood which relates time, awareness, and emotion within one framework. The consciousness state space (CSS) model is a theoretical one. It relies on a broad range of literature, hence has high explanatory and integrative strength, and helps in visualizing the relationship between different aspects of experience. Briefly, it is suggested that all phenomenological states fall into two categories of consciousness, core and extended (CC and EC, respectively). CC supports minimal selfhood that is short of temporal extension, its scope being the here and now. EC supports narrative selfhood, which involves personal identity and continuity across time, as well as memory, imagination and conceptual thought. The CSS is a phenomenological space, created by three dimensions: time, awareness and emotion. Each of the three dimensions is shown to have a dual phenomenological composition, falling within CC and EC. The neural spaces supporting each of these dimensions, as well as CC and EC, are laid out based on the neuroscientific literature. The CSS dynamics include two simultaneous trajectories, one in CC and one in EC, typically antagonistic in normal experiences. However, this characteristic behavior is altered in states in which a person experiences an altered sense of self. Two examples are laid out, flow and meditation. The CSS model creates a broad theoretical framework with explanatory and unificatory power. It constructs a detailed map of the consciousness and selfhood phenomenology, which offers constraints for the science of consciousness. We conclude by outlining several testable predictions raised by the CSS model.

## Introduction

Every human experience, those we are aware of and those we are not, is embedded in a subjective timeline, and is tinged with emotion, be it the subtlest. At the same time, each experience inevitably evokes a certain sense of self, either minimal (i.e., non-conceptual first-person content, without personal identity) or expanded and autobiographic (i.e., personal identity and continuity across time). Human experiences which are devoid of a sense of time, phenomenal awareness, and emotional tone would largely fall either into a category of neuropathology, or of an altered state of consciousness. Certainly, time, awareness, and emotion are all necessary ingredients of consciousness and selfhood. But how are these all related to each other? Undoubtedly, an endeavor relating these concepts within one framework, which bridges phenomenology and neuroscience, is a presumptuous attempt. However, this is what we will cautiously try to propose here, a model named the consciousness state space (CSS), building on current formulations of consciousness and self, supported by neuroscientific evidence.

The model is rooted in a view of the embodied mind, held by both philosophers and cognitive neuroscientists (Varela et al., 1991; Damasio, 1999; Lakoff and Johnson, 1999; Cosmelli and Thompson, 2010), suggesting that consciousness behaves like a complex non-linear dynamical system (Varela et al., 1991; Thompson and Varela, 2001; Smith, 2005; Cosmelli et al., 2007) created by a state-space (Fell, 2004; Werner, 2009). As CSS is informed by both empirical evidence from cognitive neuroscience and phenomenological accounts, it is essentially a neurophenomenological model. Importantly, CSS is a theoretical model. Yet, it relies on a broad range of literature, hence has high explanatory and integrative strength, and helps in visualizing the relationship between different aspects of experience. This is in alignment with Revonsuo's (2003) proposition: “The science of consciousness should direct considerable resources to the systematic study of phenomenological issues, in order to first construct a detailed map of the phenomenal level of description. … for the features of the phenomenal level (how it is structured, how it dynamically changes across time, and so on) offer top-down constraints for the science of consciousness in the search for potential explanatory mechanisms in the brain” (p. 3).

Briefly, CSS suggests that three dimensions, time, awareness, and emotion, create a state-space encompassing all possible total system behaviors, i.e., a repository of all potentially accessible phenomenological states. These, in turn, fall into two large categories of consciousness, each with its respective sense of self. Section A Dual Organization of the CSS describes the dual organization of the CSS, as well as its neural space. Section The Three Dimensions of the CSS describes the three dimensions of the CSS. Section The Dynamics within the CSS describes the typical antagonistic dynamic behavior of the system, as well as atypical behavior of the CSS, when the typical antagonistic relationship between the two categories is reduced, for example during the experience of flow and in meditation. In section A Comparison to Other Models of Consciousness we compare CSS to other theories of consciousness, to highlight its unique contribution. We conclude in section The Limitations, Predictions and Contribution of the CSS Model by outlining the model's limitations, as well as its contribution by providing examples of testable predictions.

## A dual organization of the CSS

### A dual organization of consciousness, self, and brain activity

While avoiding philosophical definitions of consciousness (e.g., James, 1890/1950; Searle, 1994), which are beyond the scope of this paper, the term *consciousness* here generally denotes, as in previous neuroscientific approaches (Edelman, 2006; Boly et al., 2009), an experienced property of mental states and processes, which is lost during a dreamless deep sleep, deep anesthesia or coma. Consciousness and self-consciousness are tightly related, based on both philosophical accounts and cognitive theories (e.g., Gennaro, 1996; Natsoulas, 1998; Kriegel, 2004; Morin, 2006; Gallagher and Zahavi, 2008; Damasio, 2012). We are aware that the concept of self has many definitions and that there is no consensual framework for conceptualizing the various aspects of the self. Yet, we adopt here an increasingly accepted framework for the self, grounded in James' (1890/1950) differentiation between the self as “I,” the subjective knower, a momentary enduring presence, and the self as “me,” the object that is known, the self-concept and autobiographical identity. This framework distinguishes between the “minimal self” (MS), a self that is short of temporal extension, which is endowed with a sense of agency, ownership, and non-conceptual first-person content, and the “narrative self” (NS), which involves personal identity and continuity across time, as well as conceptual thought (Gallagher, 2000). Consciousness can also be divided into a simpler and a more complex form, each one of them supporting one type of self-experience. The first is core-consciousness (CC), which supports the MS, its scope being the here and now. The second is extended-consciousness (EC), which supports the NS, and involves memory of past, imagination of future, and verbal thought (Damasio, 1999, 2012). Importantly, while CC is independent of the EC, and relies only on its exchange with the body (and environment), the EC is always dependent on CC (Damasio, 1999). Hence, the NS is dependent on the MS, but not vice versa.

In cognitive neuroscience the MS and NS have been attributed to various different neural processes. Following, we will refer to these neural spaces as N_ms_ and N_ns_, respectively. While these neural spaces cannot yet be fully identified, there is accumulated knowledge suggesting main brain regions involved. NS has been conceptualized as self-referential processing, such as assessing one's personality, appearance or feelings and recognizing one's own face or name. The neural regions supporting self-referential processing are mainly the midline regions, including the medial prefrontal cortex (mPFC), posterior cingulate cortex (PCC) and precuneus (Gusnard et al., 2001; Northoff and Bermpohl, 2004; Northoff et al., 2006), as well as the temporoparietal junction (TPJ) and temporal pole (Christoff et al., 2011). The MS has been attributed to self-specifying processing, experiencing oneself as the agent of perception, action, cognition and emotion. The cortical regions suggested to be involved include those related to sensorimotor integration (such as motor and supplementary motor area—SMA) and proprioception (the insula), as well as higher-level regulatory regions, including dorsal anterior cingulate cortex (dACC) and dorsolateral PFC (DLPFC) (Legrand, 2006; Legrand and Ruby, 2009; Christoff et al., 2011). Other regions involved in the sense of agency include the TPJ and inferior parietal lobule (IPL) (Chaminade and Decety, 2002; Blanke and Metzinger, 2009).

Intriguingly, N_ms_ and N_ns_ can be related to a dual organization of the cortex. Accumulating evidence supports this notion, showing that thalamo-cortical networks can be divided into two, often antagonistic, global systems (Fox et al., 2005; Golland et al., 2007; Tian et al., 2007; Soddu et al., 2009): (i) a system of inward-oriented networks (the “intrinsic” or default mode network - DMN); and (ii) a system of externally-oriented, sensory-motor networks (the “extrinsic” system). Resting-state activity involves the DMN (Raichle et al., 2001; Greicius et al., 2003), a task-inhibited network related to self-reference and mind-wandering. The DMN includes a consistent set of five regions that comprise the mPFC, PCC, IPL, medial temporal lobe (MTL) including the hippocampus, and lateral temporal cortex (LTC) (Buckner et al., 2008). Task-induced neural activity is related to the dorsal attention network (DAN), which includes regions in the frontal eye fields, ventral premotor cortex, the supplementary motor area (SMA), superior parietal lobule, intraparietal sulcus, and motion-sensitive middle temporal area (Corbetta et al., 2008). Interposed between them is suggested to be the frontoparietal network (FPN), which includes the anterior PFC, DLPFC, dorsomedial superior frontal, ACC, anterior IPL, and anterior insular cortex (Vincent et al., 2008). The FPN cooperates with either one of these typically antagonistic systems, possibly integrating information from, and adjudicating between, these two potentially competing brain systems (Vincent et al., 2008; Spreng et al., 2010; Smallwood et al., 2012). The FPN can be broken down into two sub-networks (Seeley et al., 2007), the “executive control network” (DLPFC and IPL) and the “salience system” (anterior insula and ACC), with the latter also being specifically attributed the role of switching between the intrinsic and extrinsic systems (Menon and Uddin, 2010). Following, we will refer to this interposed network generally as N_i_.

### The CSS contains two concentric spheres

**Figure 1:**
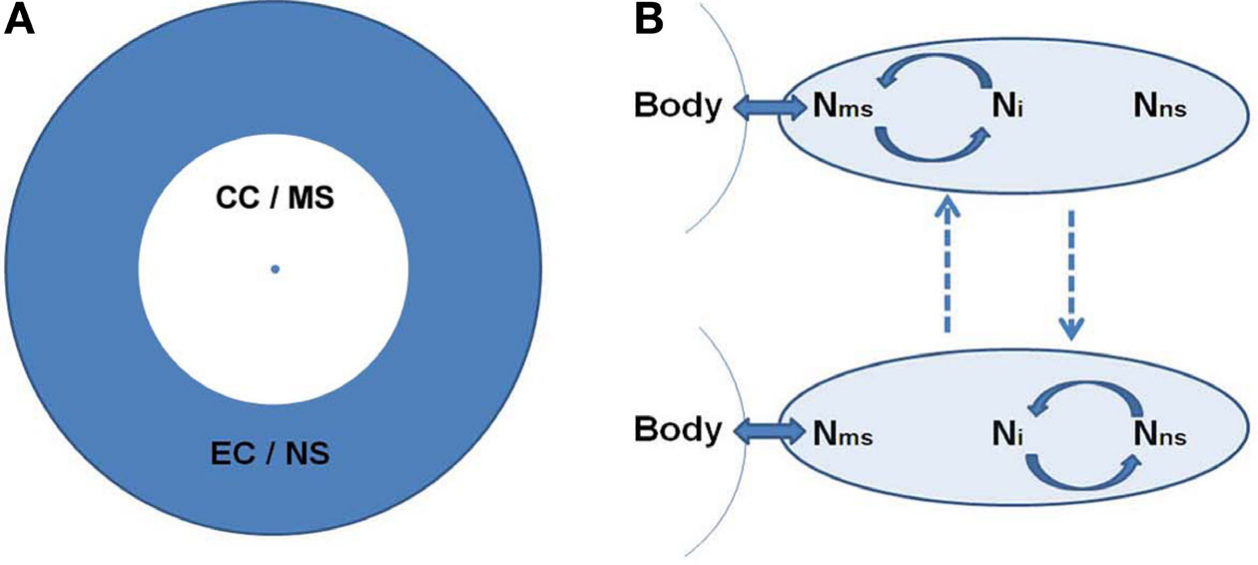
**(A)** The concentric organization of the Consciousness State Space (CSS). The central point denotes the body. Around it is the core-consciousness (CC) and minimal-self (MS) sphere in white, surrounded by the extended-consciousness (EC) and the narrative-self (NS) sphere in gray. This circular organization depicts a shorter and longer psychological distance from the body for the CC/MS and EC/NS, respectively. **(B)** The suggested neural space, depicted as an oval. Within it we identify three main networks, two of them supporting the MS and NS (N_ms_ and N_ns_, respectively), and the third interposed between them (N_i_). The body's condition becomes available to the neural space through N_ms_. Ni switches between synchronization (marked by circular arrows) with the two other networks. Dashed lines denote alternation between the neuronal states. The top state corresponds phenomenologically to CC/MS, and the bottom to EC/NS. For the suggested brain regions for each neural space see section A Dual Organization of Consciousness, Self, and Brain Activity.

Based on the dual organization of consciousness, self, and underlying neural activity, CSS is organized into two concentric spheres around the body. The concentric organization depicts the reliance of each sphere on the previous level: CC relies on the body, while EC relies on CC. The inner sphere of CC/MS is phenomenologically related to the body, experiencing agency and momentary sensory experiences. It is embodied. The inner sphere is surrounded by the EC/NS sphere, which is phenomenologically further away from the body, in the realm of conceptual thought, language, memories and imagination, and relies more on mental representation, rather than actual sensory experiencing (Figure 1A).

We refer more generally to the neural space, mainly building on current neuroscientific knowledge. As to the neural space of the CC/MS (N_ms_), this is the point where the physiological condition of the body, which is in constant exchange with the environment, becomes available to the brain. This mainly relies on sensori-motor integration, i.e., convergence of action and perception, allowing one to perceive the sensory consequences of one's action through action monitoring, and proprioception—perceiving the body state. The proposed regions of the brain involved in sensori-motor integration are SMA and pre SMA (Legrand, 2006; Ferri et al., 2012), while proprioception involves the somatosensory and insular cortex (Craig, 2002, 2009) as well as a deep portion of the posterior cingulate (Parvizi et al., 2006; Damasio and Meyer, 2009). The neural reference space of the EC/NS (N_ns_) is largely suggested to involve the DMN. It should be noted, however, that some posterior regions of the DMN, including the IPL and precuneus, are argued to be involved in both NS and MS due to their roles in agency (Chaminade and Decety, 2002) and CC (Damasio and Meyer, 2009), respectively. These regions can be viewed as a mutual reference space for both spheres. Between the N_ms_ and N_ns_ there is the interposed Ni, largely related to the control FPN system, which shifts between collaboration with both (Figure 1B).

Another important feature of the CSS is the existence of two simultaneous trajectories of experience, each in one of the spheres. This will be further developed in sections Second Dimension of the CSS—Awareness and The Dynamics within the CSS.

## The three dimensions of the CSS

A number of theories have described aspects of consciousness within a state-space paradigm, choosing as dimensions different parameters describing the system's behavior. For example, Allan Hobson and colleagues (Hobson et al., 2000) have introduced a three-dimensional state-space model for the classification of mental states during sleeping, dreaming and wakefulness. The dimensions of this model are activation (the information processing capacity of the system), information flow (the degree to which the information processed comes from the outside world and is or is not reflected in behavior), and mode of information processing (the way in which the information in the system is processed). This model shows how alertness, drowsiness and sensory restriction are located within the state-space in relation to each other, and suggests the underlying brain structures and chemistry. Another example was proposed by Wackermann (1999) who developed a three-dimensional global approach to representing the electrical activity of the brain. This model's dimensions are: Σ (a measure of global field strength, reflected by the data cloud in the state space, in μV), Φ (a measure of global frequency of field changes, reflected by the density of distribution of the momentary states along the trajectory speed of field change, in Hz), and Ω (a measure of spatial complexity, implying the simplicity/complexity of the data cloud, dimensionless). Using this three-dimensional complexity description, the electrical signature of a brain's macro-state may be represented by a trajectory in a three-dimensional space, which facilitates electrophysiological data reduction. More closely related to the current model, Fell (2004) suggested a three-dimensional state-space that characterizes states of phenomenal awareness, with the three state dimensions being the amount of synchronized electrophysiological activity within different frequencies. In this model, phenomenal awareness is related to decreased delta and alpha and increased gamma activity.

**Figure 2:**
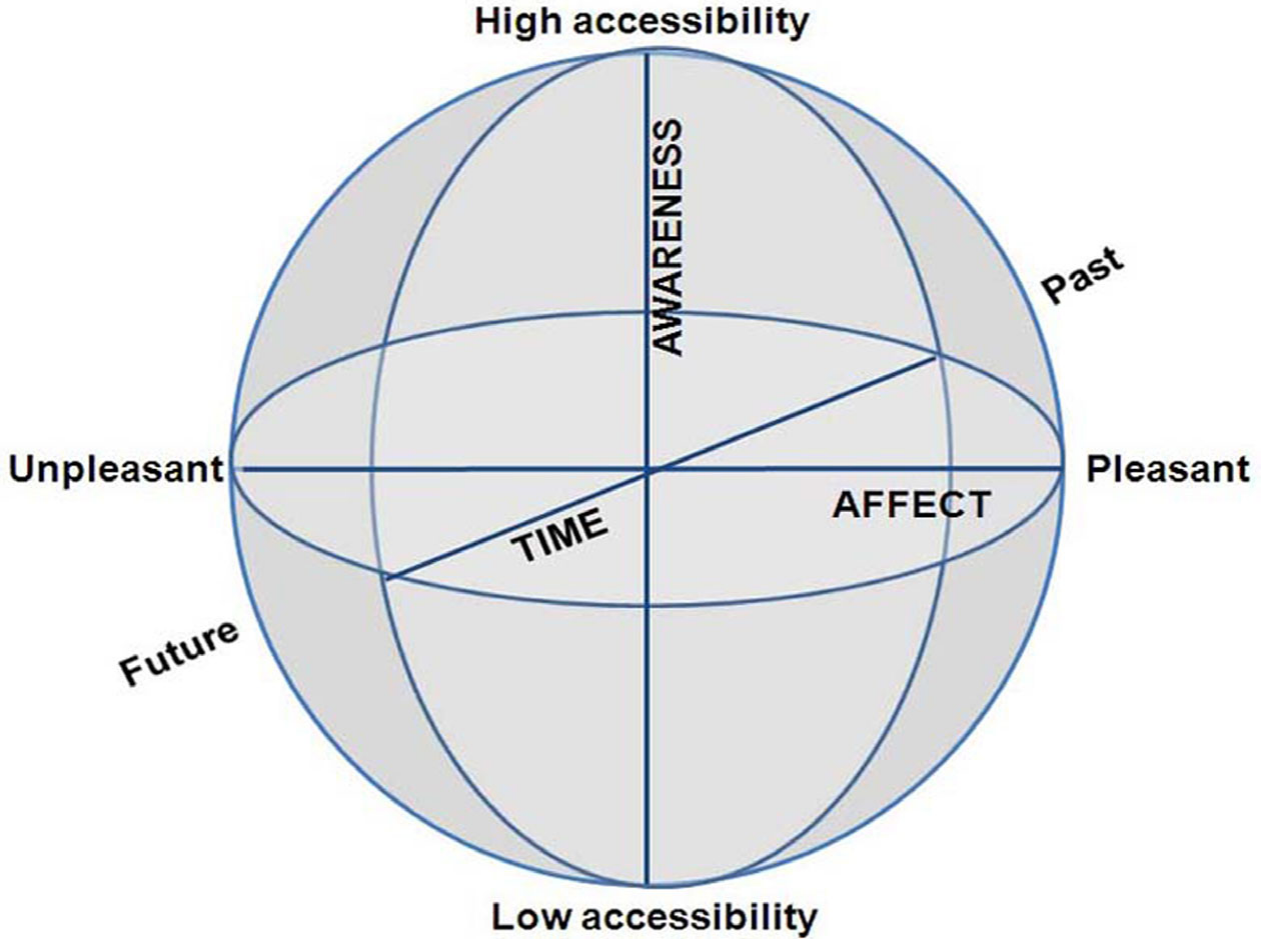
**The Consciousness State Space (CSS), depicting a phenomenological space with three psychological dimensions**.

We argue that a CSS is created by merely three phenomenological dimensions: (i) subjective time—at one end past and at the other future; (ii) awareness—at one end high and at the other low phenomenal access; and (iii) emotion—at one end pleasant and at the other unpleasant (Figure 2). Whereas the other state-space models are quantifiable—that is, the dimensions are continuous and lend themselves to quantification—the CSS model employs dimensions, which refer to psychological distance from the body. Further, the two concentric spheres of CC/EC and MS/NS, are both structured by this same 3D coordinate system, as outlined in length for each dimension separately. Pointing out these particular dimensions might raise the question as to whether, and how, other important phenomena can be manifested in terms of these three particular dimensions. We answer these questions by three arguments.

Firstly, we argue that these three dimensions are phenomenologically distinct. This is not to say that we think of these dimensions as being self-sufficient for consciousness. We fully acknowledge the crucial role of interconnectivity among these phenomenological aspects to create the unity of consciousness (Searle, 1994; Dainton, 2006). Furthermore, occasionally these dimensions rely on similar, non-specialized, brain regions (Pessoa, 2008). Yet, these dimensions can be characterized as being phenomenologically distinct, while other phenomena could be considered to result from the interplay among these three dimensions. A major example is the general construct of cognition, which includes learning, memory, thinking and language (Mayer et al., 1997), and which is also closely linked to emotion (Pessoa, 2008), hence its different facets are spread over the entire CSS.

Secondly, there are afferent and efferent functions, which are tightly related to, but occur without, consciousness. This includes action (motor output) and language, as speech (either mental or executed), which can also be considered as an action (Jones and Fernyhough, 2007). Since one becomes aware of one's action only after it has been initiated (Libet, 1985), and has sensory consequences (Frith et al., 2000; Legrand, 2006; Carruthers, 2009), these functions are excluded from CSS.

Thirdly, some key mental functions might “overlap” with, or be closely related to, the dimensions presented here. For example, attention is closely related to the awareness dimension, and is captured by it, as will be explained in the section devoted to awareness. Another example is spatial cognition: while there is much evidence which tightly links this to temporal cognition, both phenomenologically and in the neural space (Barsalou, 1999; Glicksohn, 2001; Boroditsky and Ramscar, 2002; Walsh, 2003; Glicksohn and Myslobodsky, 2006; Casasanto, 2008; Srinivasan and Carey, 2010), there is also substantial research arguing that spatial perception is actually the more fundamental dimension (e.g., Srinivasan and Carey, 2010). However, CSS depicts human experience, wherein subjective “time traveling” is far more frequent compared to “space traveling.” Our life memories are ordered along a time-line, and not a space-line (Wheeler et al., 1997; Markowitsch, 2003). For that reason, CSS includes time as one of its dimensions, and not space.

Next we describe the three dimensions of CSS in detail. Importantly, each dimension behaves differently in the two spheres, in both phenomenology and its neural space, as subsequently outlined.

### First dimension of the CSS—time

Consciousness would be inconceivable without temporality, as time is an omni-present structural feature of consciousness (James, 1890/1950). As James wrote: “The knowledge of some other part of the stream [of consciousness], past or future, near or remote, is always mixed in with our knowledge of the present thing…. These lingerings of old objects, these incomings of new, are the germs of memory and expectation, the retrospective and the prospective sense of time. They give that continuity to consciousness” (p. 606–607). Past or future events can be activated in experience voluntarily, and this constant mental time travel aids one in understanding the meaning of present happenings: “I don't simply exist in the present and happen to have the capacity to envisage the future and remember the past. Rather, human reality is characterized by a kind of temporal stretch. The past continually serves as the horizon and background of our present experience, and when absorbed in action, our focus, the center of our concern, is not on the present, but on the future goals that we intend or project” (Gallagher and Zahavi, 2008, p. 86).

We claim that this time dimension is sufficient, and can also account for the spatial aspect of experience. First, at any time point, one sees the world from only one spatial perspective (Revonsuo, 2003). Second, each episodic memory has one (and only one) spatiotemporal content (Russell and Davies, 2012). Third, the phenomenal fields of different modalities are spatially and temporally integrated, so that different features belonging to the same object are realized in the same location and time (Fingelkurts et al., 2010). Thus, spatial experience is fully integrated with temporal experience in three important ways.

**Figure 3:**
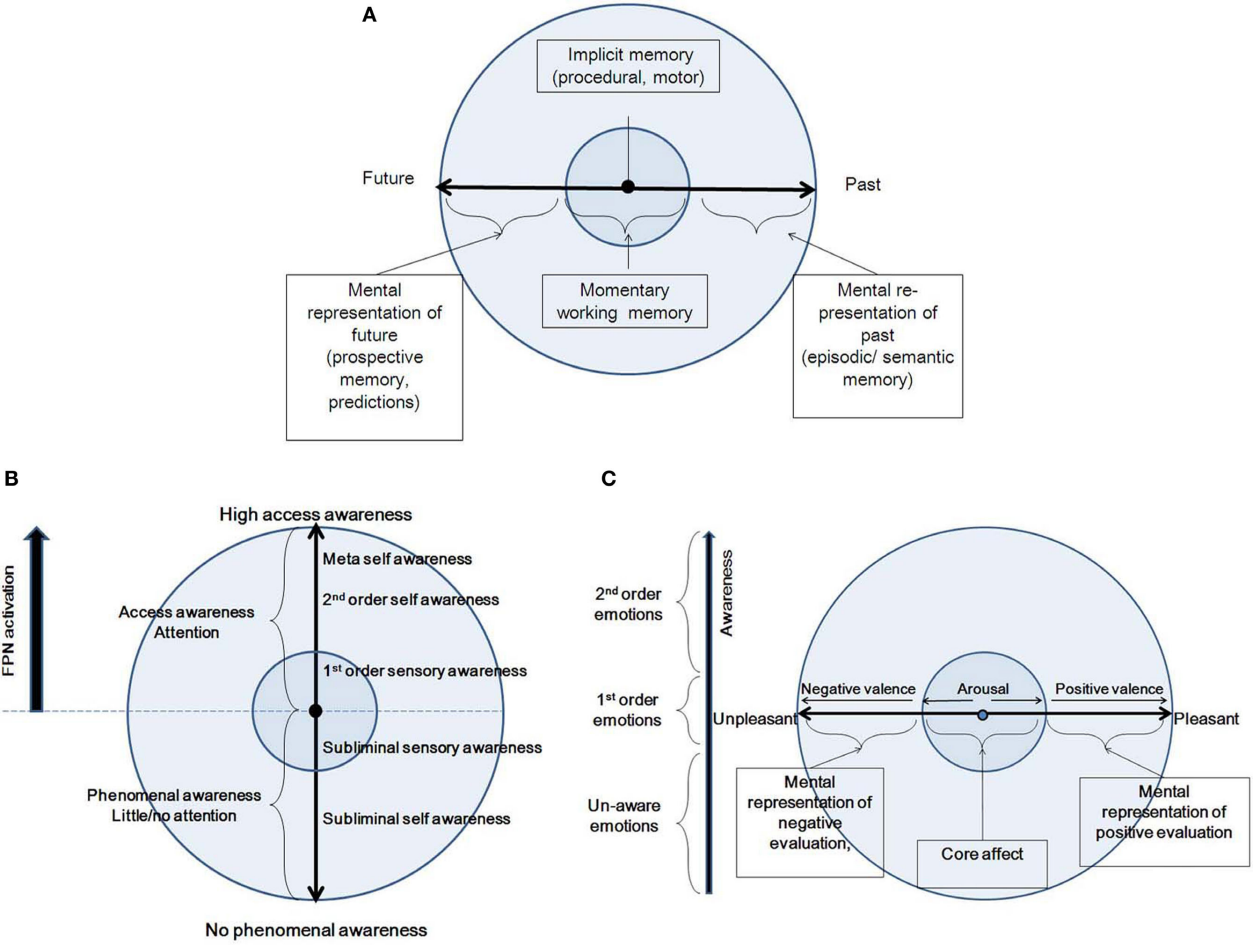
**A summary of the features of the three CSS dimensions: Time (A), Awareness (B) and Emotion (C)**.

Several cognitive models have been proposed for time-consciousness, most of them variants of a pacemaker–accumulator clock, where experienced duration is represented by a pacemaker which produces a series of pulses recorded over a given time span (Zakay and Block, 1997; Glicksohn, 2001). Yet, competing cognitive models propose that memory decay processes are involved in time perception (Wackermann and Ehm, 2006). A contrasting view to the cognitive models is Varela's (1999) dynamic model of the experience of time, according to which three different scales of duration contribute to a cognitive act: the elementary (10–100 ms), integration (0.5–3 s) and narrative (>10 s) scales. Neurophysiologically, the first two correspond to neuron-level electrophysiology and synchronization. These further correspond to the experienced present. Here, we adopt a dynamic view, akin to Varela's view. However, we differentiate between two time scales, combining Varela's elementary and integration scales into one: the immediate perception of the present moment (<3 s; see Pöppel, 1997), contrasted with the longer time scale. The longer time scale refers to the re-presentation of experience, while the second refers to the immediate perception of the present moment (Figure 3A). By “re-presentation” we mean that experience, with its full-blown, present-moment, multi-dimensional vividness, is being “projected,” or re-presented (and not represented) into another subjective time, either past or future. The fact that we usually cannot both recall and be “here and now” simultaneously is manifested by placing each of the two phenomenological categories into the two different spheres of CSS. Following, we describe in more detail these two categories along the time continuum.

The first temporal category refers to the longer time scale, and involves the re-presentation of experience in the past and in the future. Unlike immediate perception, this is psychologically further away from the body: when one's conscious awareness re-lives the past or the future, one's conscious awareness is decoupled from the body (which experiences the now). It encompasses mental re-presentations of other “nows,” relived or imagined. The intriguing ability of the human mind to mentally travel through time, enabling one to relive past experiences through memory, or project oneself into the future by generating a prediction based on memory, has been also referred to as autonoetic (self-knowing) consciousness (Wheeler et al., 1997; Stuss et al., 2001; Markowitsch, 2003). Hence, this experience pertains to the NS, and is within the EC/NS sphere. At the past end, we find re-presentation of the far or near past, by means of retrospective memory retrieval. This includes either true or false memories, which are experienced as being phenomenologically similar (Lampinen et al., 1997). Here, we adopt Conway's phenomenological description of autobiographic memory (Conway and Pleydell-Pearce, 2000; Conway, 2009), as well as Morin's (2006) conceptualization of long-term memory. Conway relates autobiographic memory with the NS (Conway, 2005). Autobiographic memory consists of recollected episodes from one's life, which are based on both episodic as well as semantic memory (Tulving, 1985; Conway and Pleydell-Pearce, 2000; Conway, 2005). Autobiographic knowledge comprises several levels of categorization. First is event-specific knowledge, which is a summary record of sensory-perceptual–conceptual-affective processing derived from working memory, which is predominately re-presented in the form of visual images (for example, a specific restaurant). Event-specific knowledge, in turn, is contextualized within a general event (e.g., during the vacation in Greece). The general event, in turn, is associated with one or more lifetime periods that locate the more specific knowledge within an individual's autobiographical memory as a whole (e.g., it was just after our marriage) (Conway and Pleydell-Pearce, 2000; Conway, 2005). What is common to all these levels of autobiographic memory is the phenomenal feeling of remembering: “the feeling signals the state in an experiential way. Recollective experience, the sense of the self in the past and the episodic imagery that accompanies that sense, indicate to the rememberer that they are in fact remembering and not daydreaming, fantasying, or in some other non-memory state” (Conway, 2005, p. 614). At the other end of the time continuum, the future, we find prospective memory, which is memory for future intentions (Glicksohn and Myslobodsky, 2006), and refers to the functions that enables a person to carry out an intended act after a delay (Burgess et al., 2001). Another ubiquitous phenomenon is the generation of predictions and future simulations based on previous autobiographic knowledge (Schacter and Addis, 2007; Schacter et al., 2007), sometimes termed “proaction” (Bar, 2007, 2009). Mental time-travelling to the future and the past can be actually “experienced” in the “here and now” (Gilbert and Wilson, 2007): if the mental simulation is strong enough, the imagined or recollected images can evoke bodily reactions. At the same time, actual sensory stimulation from the environment is blocked, and one experiences sensory decoupling from the environment (Smallwood et al., 2007).

We now turn to the second category, the immediate perception of the present moment (<3 s), which pertains to the CC/MS sphere. Various models for the phenomenology of immediate time perception have been proposed (Dainton, 2008). One major category is retentional models: our experiencing of change occurs within episodes of consciousness which themselves lack temporal extension, but whose contents coordinate with past and future by virtue of their place in the temporal structure (Dainton, 2008). Specifically, we adopt the retentionalist model for consciousness of time outlined by Gallagher and Zahavi (2008), which assumes a Husserlian view: the immediate sensation, or the “primal impression” is combined with retention (being aware of the “just-passed” slice of the experience) and protention (being aware of the “just-about-to-be”). A perception cannot merely be a perception of what is now, but must include a retention of the just-passed and a protention of what is about to occur. Importantly, retention and protention are not memory, or imagination, which re-present the experience. Rather, they are actual experience. Unlike long-term memory and expectation, they are involuntary and automatic processes, and they could be argued to be working memory (Vogeley and Kupke, 2007; Gallagher and Zahavi, 2008). This experience is related in our model to the MS.

Turning to the neural space, the first category of autonoetic consciousness, within the EC/NS sphere, should involve the N_ns_. In contrast, CSS predicts that the immediate perception of the present, within the CC/MS sphere, is related to the N_ms_ and bodily processing. As subsequently presented, these predictions are confirmed by neuroscientific evidence.

The most important neural structure for memory is the hippocampus, the locus of interaction between working memory and long-term memory (Fell and Axmacher, 2011). It has been proposed that memory initially depends on the hippocampus. However, with increasing time, the hippocampus becomes less important, and the involvement of multiple cortical regions increases, including the medial frontal gyrus and precuneus (Smith and Squire, 2009). Yet, new findings confirm the important role of the hippocampus even in retrieval of long-term, established memories, in collaboration with the ACC (Suzuki and Naya, 2011). In a nutshell, the hippocampal complex is essential for encoding, retaining, and recovering experiences, enabling the immediate subjective and vivid experience. Other regions, mainly the prefrontal cortex, select, organize, help retrieve, monitor, and verify the hippocampal recollection (Moscovitch, 2008). Though both recent and remote memories are associated with hippocampal activation, it was found that activations associated with more recent memories cluster at the anterior hippocampus, whereas those associated with more remote memories are distributed across its length (Gilboa et al., 2004). Not only memory, but also planning involves the hippocampus, as well as frontal and parietal structures. Strikingly, there is an overlap between memory systems and the network involved in foresight, and these two overlapping regions also overlap with the DMN (key component in N_ns_), including the hippocampus, mPFC, precuneus and lateral parietal cortex (Bar, 2007, 2010; Schacter and Addis, 2007; reviewed by Schacter et al., 2007). Another line of research, on mental time traveling and “self-projection,” revealed the involvement of the IPL (Nyberg et al., 2010), and the temporo-parietal junction (Arzy et al., 2009), which are key regions for self-referential processing and which are considered components of the DMN.

Turning now to the cortical regions, which have been suggested to be involved in the immediate perception of the present moment, Rubia and Smith (2004) emphasize the DLPFC, ACC, SMA, and IPL in their review of the literature. The IPL is also strongly suggested by others who review the literature (Walsh, 2003; Oliveri et al., 2009). Another suggested region is the insula (Craig, 2002, 2009; Wittmann, 2009), relating cognition of duration with proprioception. This proposition was supported by a functional magnetic resonance imaging (fMRI) study, showing a linear build-up of neuronal activation in the insula during a time reproduction task (Wittmann et al., 2010) and by an anatomical study (Gilaie-Dotan et al., 2011), showing that the gray matter volume of the right sensory cortex is correlated with the ability to discriminate time intervals. In addition to the literature on time perception, we consider Baddeley's (1992, 2003) influential model of working memory. Here, again, we find that the DLPFC plays an important role, as an executive control system, assisted by two subsidiary storage systems: the phonological loop and the visuospatial sketchpad (including right and left IPL and premotor cortex, respectively), both of which store perceptual information. Indeed, the DLPFC is believed to provide a buffer to hold information in mind, and to order it in space-time (Dehaene and Naccache, 2001). To conclude, as hypothesized, all the neural regions that are related to momentary experience of time, as well as to working memory, are within the FPN and DAN, considered as key elements in N_ms_ and N_i_, and in contrast, autonoetic consciousness and prospective memory involves the DMN, as a key element in N_ns_.

### Second dimension of the CSS—awareness

Awareness is a primary feature of consciousness, being the subjective experience of internal phenomena, a perception of the field of inner and outer events that encompasses one's reality at any given moment, the state of perceiving (Laureys, 2005; Cohen and Dennett, 2011). Awareness can be largely categorized into two types, following the influential conceptualization of Block (1995, 2007), represented in CSS as the two sides of the awareness dimension. The first is access awareness, which corresponds to states that can be reported on, by virtue of high-level cognitive functions such as memory and decision making, and which necessitates attention. The second is phenomenal awareness, related to private first-person experience, and occurs without—or with very little—attention (Kouider et al., 2010). In contrast to this division, awareness could also be conceived of as a graded phenomenon (Kouider et al., 2010): at one end expanded awareness, when all levels of relevant processing are accessible, and at the other end complete non-awareness, when all levels of processing are not accessible. Intermediately, there is partial awareness, combining awareness at some level and unawareness at another level of processing.

A possible solution to the current debate whether top-down attention is necessary for consciousness (Cohen et al., 2012a,b) or if these are two independent processes (Koch and Tsuchiya, 2007a,b; Tsuchiya et al., 2012) could be settled if we consider that phenomenal awareness can emerge without top-down attention (Aru and Bachmann, 2013), in contrast to access awareness. This would support the notion that attention allows information to be more fully transmitted across cortical regions than unattended information (Cohen et al., 2012b), hence is required for access awareness. This argument supports our proposition that the awareness dimension stands for attention as well.

In the following, we describe various phenomenal states along the awareness continuum, moving from no phenomenal access to high phenomenal access (Figure 3B). In doing so, we largely rely on Gallagher and Zahavi's (2008) account of pre-reflective and reflective consciousness, as well as on Morin's (2006) social/personality model, describing degrees of consciousness based on several theories (including Brown, 1977; Natsoulas, 1998; Schooler, 2002). Starting at no phenomenal access, we find outside CSS states of consciousness in which there is no phenomenal awareness to either external or internal input. These include the dreamless portion of deep sleep, coma, anesthesia, vegetative state, epileptic loss of consciousness, and somnambulism. When one regains awareness, there is still no access or memory as to external or internal happenings during that state. Further along the continuum, there are preconscious states and subliminal experiences. A division should be drawn between inaccessible internal and external input. Processing of internal input is conducted within the EC/NS sphere, and is referred to here as “subliminal awareness.” This includes normal states of day dreaming, as well as pathological states such as dissociation. Processing of external input is conducted within the CC/MS sphere, and is referred to as “subliminal sensory awareness.” These states include the natural decoupling of attention from sensory processing (Smallwood et al., 2007), as can occur during driving. All of these states are not accompanied by top-down attention.

Next, we describe the access states along the awareness axis, which can be conceptual or non-conceptual (Kapitan, 2006), as subsequently detailed. First, within the CC/MS sphere, there is first-order awareness, also called pre-reflective awareness (Gallagher and Zahavi, 2008). This is an implicit and direct awareness to experience, prior to any reflection on the experience. In this state, according to Morin (2006), one will directly be attentive and process external input from the environment, without conceptual elaboration of the mental events that are taking place. Hence, the organism will be totally immersed in experience. These states are accompanied by top-down attention to external input (Chun et al., 2011). States along the access awareness within the EC/NS sphere involve second-order awareness, also called reflective awareness (Gallagher and Zahavi, 2008). This is an explicit, conceptual, and objectifying awareness, which is accompanied by focal attention to internally generated input (Chun et al., 2011). In this state, one attends directly to the cognitive experience itself. It is described by Morin (2006) as the capacity to become the object of one's own attention, a process that occurs when an organism focuses not on the external environment, but on the internal milieu. In its extreme form, it becomes meta-awareness, being aware that one is aware (Morin, 2006).

**Figure 4:**
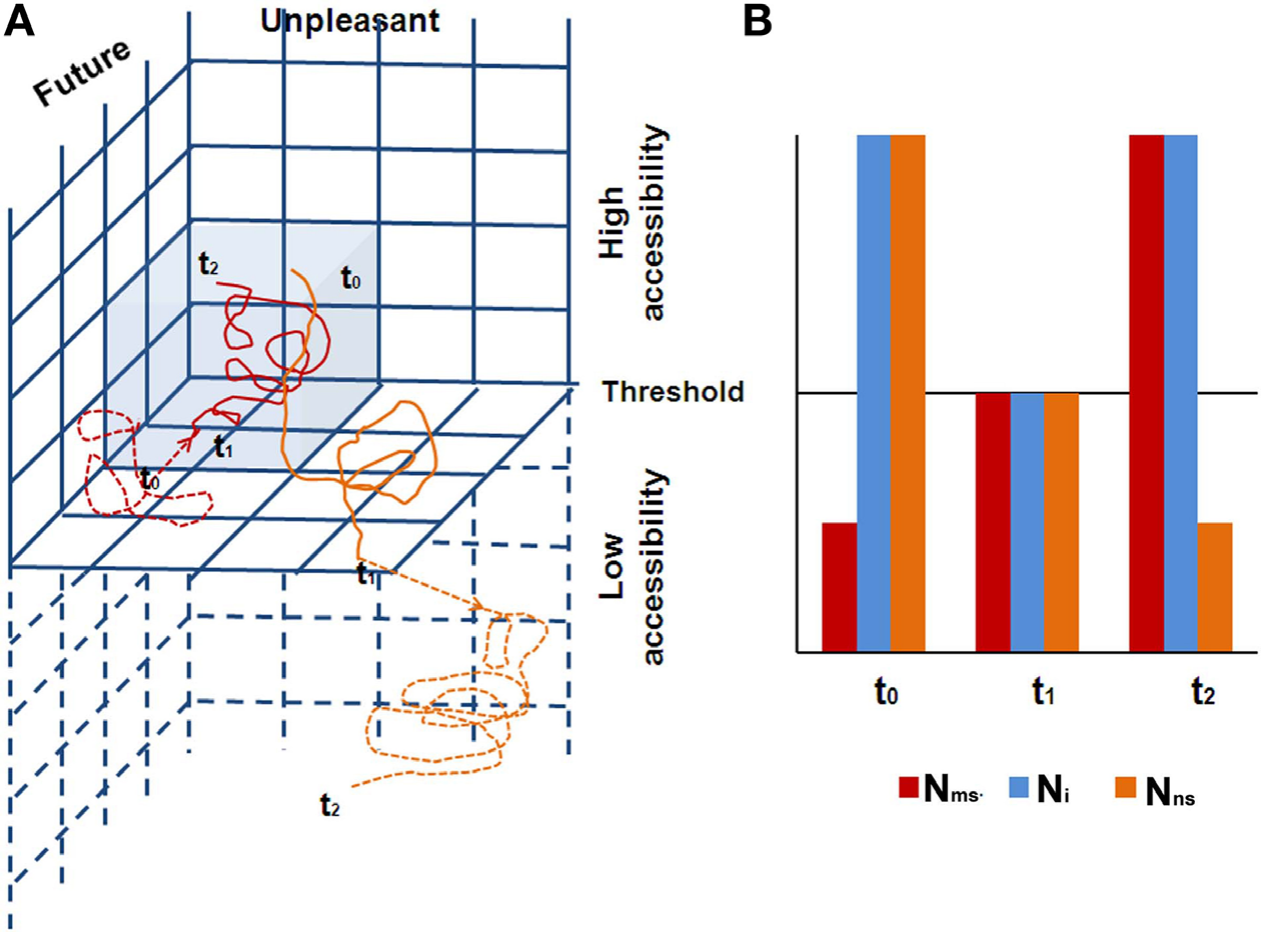
**(A)** The two trajectories in the CSS are shown (red denotes the CC/MS trajectory, and orange the EC/NS trajectory), with their dynamic behavior. A full line denotes access awareness, while a dashed line denotes phenomenal awareness. The figure shows a quarter of the CSS, depicting the future/unpleasant space. Gray denotes the CC/Ms 3D space. The three time points demonstrate the typical antagonistic behavior of the two trajectories. At t_0_, the CC/MS trajectory is under the threshold for access awareness; hence one has access awareness to the EC/NS trajectory. For example, one is driving, decoupled from sensory awareness (present moment and relatively neutral emotionally) while mind wandering, imagining a future unpleasant experience. At t_1_ there is a threat on the road, resulting in a phase transition: the CC/MS trajectory arrives at the threshold for access awareness, and at the same time, the EC/NS trajectory shifts under the threshold for access awareness. At t_2_, the CC/MS trajectory expresses negative arousal due to the danger, while the EC/NS trajectory becomes even more unpleasant due to self-criticism for lack of awareness to the driving, continuing at a subliminal level of awareness; **(B)** a schematic diagram of the neural space behavior over the same specific three time points. N1 corresponds to the neural space of the CC/MS sphere, and N2 to the neural space of the EC/NS sphere. At t_0_, FPN is collaborating with N2 (one phenomenally experiences thoughts). At the short transition at t_1_, all networks are at threshold level for access awareness (one phenomenally experiences “no thoughts”), and at t_2_ the FPN collaborates with N1 (one phenomenally experiences high concentration while driving).

We rely on neuronal global workspace theory (Dehaene and Naccache, 2001; Dehaene et al., 2006) to describe the corresponding neural space, suggesting that conscious access is produced through the interaction between specialized neural subsystems and a multimodal limited capacity global workspace (Baars, 2002, 2005), the FPN (the key component of N_i_). During states of phenomenal awareness, including subliminal and preconscious states, activation due to internal or external input does not involve the FPN. In contrast, during states of access awareness, synchronized activity increases in the FPN, which becomes capable of guiding intentional actions including the production of verbal reports. The transition between phenomenal and access awareness is sharp, as expected in non-linear dynamic systems (Dehaene et al., 2006). Furthermore, CSS posits two simultaneous trajectories, one in the EC and the other in the CC. As these two trajectories are usually antagonistic, habitually one has access awareness only to the phenomenology related with one of the trajectories, while the phenomenology related with the second trajectory continues its activity at a sub-threshold level, within the phenomenal awareness continuum (Figure 4). Hence, the brain alternates dynamically, shifting awareness from internal to external processing. The actual phenomenal experience is constantly dictated by the neural space that is more active at the moment, and which is synchronized with the N_i_. When one is immersed in “intrinsic” (i.e., N_ns_) activity, one is “decoupled” from extrinsic processing (i.e., N_ms_), which nevertheless continues (Smallwood et al., 2007; Vanhaudenhuyse et al., 2011). And vice versa, while externally engaged, the intrinsic system is continuously activated in a “sub-threshold for awareness” manner. Indeed this is what Singer has been proposing from the early 60s, in his exposition of daydreaming (Singer, 1966). As he has more recently suggested (Singer, 2009, p. 196), “Many years ago I proposed that what we now call the brain's default network may be almost continuously active at a subthreshold level.” The FPN alternates between cooperation with the intrinsic or the extrinsic systems. This notion is supported by the findings that a systematic impairment of FPN was found in altered states of consciousness, such as sleep, anesthesia, coma, vegetative state, epileptic loss of consciousness, and somnambulism (summarized by Boly et al., 2009), all of these being states which are considered outside the model's awareness dimension. This suggests that an intact FPN, enabling access awareness, is a prerequisite for a normally functioning CSS.

In relation to this neural space, both second-order awareness (within the EC/NS sphere, upper side of continuum) and mind wandering, i.e., subliminal awareness (within the EC/NS sphere, lower side of continuum) have been related to DMN activity (N_ns_). However, what differentiates between states has been shown to be DLPFC activation, which characterizes thoughts that occur with access awareness (Smith et al., 2006; Christoff et al., 2009). The suggested role of FPN as switching collaboration between the two neural spaces which support the two phenomenological spheres along the access awareness is illustrated by the following studies. In relation to the inner sphere of CC/MS, an fMRI study (Ferri et al., 2012) showed that the “bodily-self” (self rooted in bodily motor experience) recruits pre SMA, SMA and insular regions, belonging both to the FPN and DAN systems (N_i_ and N_ms_, respectively). In relation to the outer sphere of EC/NS, an fMRI study emphasized the importance of the DLPFC (N_i_), as participants learned to regulate its activation by turning their attention toward and away from the contents of their own thoughts, or their DMN-intrinsic (N_ns_) system (McCaig et al., 2011).

### Third dimension of the CSS—emotion

Emotion and consciousness are considered by many to be inseparable (e.g., Damasio, 1999; Lambie and Marcel, 2002; Panksepp, 2005; Barrett et al., 2007; Tsuchiya and Adolphs, 2007), as each conscious state is endowed with some form of emotion, to the point that even the perceptual representation of everyday objects carries subtle affective tone (Lebrecht et al., 2012). Put in the words of Searle (1994, p. 7), “part of every normal conscious experience is the mood that pervades the experience. It need not be a mood that has a particular name to it, like depression or elation; but there is always what one might call a flavor or tone to any normal set of conscious states.”

Emotion states are generally agreed to bear two important phenomenal features, the one is mental and the other bodily, grossly speaking. Hedonicity is both intrinsic to bodily states, and depends on the interpretation placed on them. A similar differentiation has been done with many, albeit calling it by various names. Schachter and Singer (1962) described emotional experience as a combination of general arousal, and the cognitive attribution of the cause of this arousal. Similarly, Mandler (1984) distinguished between non-specific arousal, awareness of which provides the intensity of the emotional experience, and the evaluative structure (cognitive interpretation of the situation), which provides the particular content and quality of emotional experience. Damasio (1999) views emotional experience as consisting of sensory changes that occur in the viscera and internal milieu (which he calls emotions) and the mental image of these sensory patterns (which he calls feelings). According to Lambie and Marcel (2002), any emotional state is defined by a combination of two things: the bodily action readiness (and its representation) and the evaluative description (a mental representation). The first includes certain bodily and brain systems which are activated in response to stimuli (chiefly the limbic, autonomic, hormonal and aspects of the skeletal nervous system). The second, the evaluative description, is an appraisal leaving a record, a description of how one's concerns or one's self have been affected by the event. This is a mental representation. Barrett's (Barrett, 2006; Barrett et al., 2007) view of emotion involves two operations. The first is called “core-affect,” which includes bodily fluctuations that are represented in the brain. The second is “conceptualization,” a process by which stored representations of prior experiences (i.e., memories, knowledge) are used to make meaning out of sensations in the moment. To summarize, there is general agreement that emotional states bear two important phenomenal features, one mental and the other bodily. Following, we will refer to these two components of emotion as valence (a subjective feeling of pleasantness or unpleasantness) and arousal (extent of bodily excitation), respectively.

We suggest that the manifestation in CSS of these two phenomenological qualities of the emotional experience, namely arousal and valence, is within the CC/MS and EC/NS spheres, respectively (Figure 3C). Within the CC sphere, arousal increases in two directions stemming from a minimal degree of arousal at the center of the emotion continuum. Valence, manifested within the EC sphere, increases toward the pleasant and unpleasant ends of the continuum. Each emotional experience has an arousal component, namely a bodily and sensory element, and valence, or a mental representation, in line with the above formulations of the phenomenology of emotions, and with the two CSS trajectories. The relationship between valence and arousal has been subject to a long debate, and various models have been proposed. A classical description is Russell's (1980) circumplex, suggesting an orthogonal relationship. In another framework, Lewis et al. (2007) suggest that aspects of valence generate a U-shaped curve with arousal. According to such a framework, strong positive valence is accompanied by strong positive arousal, and similarly for the negative aspects. Yet, others suggest that the dissociation between valence and arousal might be an issue of measurement more than reflecting distinct qualia underlying emotional experience (Larsen et al., 2003; Kron et al., 2013). For example, Russell (1989) has explicitly opposed the idea that arousal is only a physiological concept, writing (p. 106), “… there is no more reason to speak of arousal as strictly physiological and pleasure-displeasure as strictly mental then there is to express it the other way around.” In comparison to the ongoing debate, the CSS model accounts for both valence and arousal, and allows for an orthogonal relationship between them. This means that each point on the arousal continuum could be in principle accompanied by any simultaneous point on the valence continuum, as it depends on the interpretation placed on it, which might be multiple for any given bodily state (as accounted by Schachter and Singer, 1962). As for the Lewis et al. U-shaped proposed relationship, we propose this should be rejected because negative arousal can sometimes be accompanied by positive mental evaluation, as in Schachter and Singer (1962), or as in the example of watching safely a horror movie. Further, the interplay between the emotion and awareness dimensions within CSS predicts a novel relationship between arousal and valence, namely antagonism: emotional states involve both evaluation and the products of arousal, but both of these need not be simultaneously present in experience or awareness, and certainly are not always experienced as such. This means that one can have access awareness to either the arousal or valence aspect at each specific moment and state, and that access awareness alternates between them. As a trait, one can be more prone to emphasize either the arousal or the valence, as shown by Barrett (1998). In addition, the interplay between the emotion and awareness dimensions creates a wide spectrum wherein emotional experience can be classified in terms of accessibility, as suggested by Lambie and Marcel (2002), Damasio (1999) and Frijda (2009), from non-conscious emotions, through phenomenal emotions (1st-order emotional experience), to awareness of emotional experience (2nd-order, emotional experience).

CSS predicts that the neural space for the arousal component of emotion should be related to the N_i_ and N_ms_ (their key components being DAN and FPN, respectively), while the valence component should be related to the N_ns_ (i.e., DMN). The evidence for that is based on a meta-analysis conducted by Barrett and colleagues (Barrett et al., 2007; Kober et al., 2008; Lindquist et al., 2012), stemming from a constructionist approach to emotion, where the assumption is that emotional mental states result from an interplay of more basic psychological processes that may not, themselves, be specific to emotion (Barrett et al., 2007; Kober et al., 2008; Lindquist et al., 2012). According to this meta-analysis, the distributed network involved in realizing core-affect includes several sub-cortical, as well as cortical regions: the amygdala, which signals whether exteroceptive sensory information is motivationally salient; the anterior insula, which plays a key role in representing core affective feelings in awareness based on its role in the awareness of bodily sensations and affective feelings; portions of the orbitofrontal cortex, as a site that integrates exteroceptive and interoceptive sensory information to guide behavior; ACC, and more specifically subgenual ACC, regulating somatovisceral states, pregenual ACC, as a visceromotor (i.e., autonomic) control area involved in resolving which sensory input influences the body when there are multiple sources of sensory input, and anterior midcingulate cortex (a part of the FPN), delivering sources of exteroceptive and interoceptive sensory information to direct attention and motor response. The regions suggested by this constructionist approach fall largely within the interoceptive/exteroceptive processing network, as well as the FPN, as suggested by CSS.

In Barrett's (Barrett et al., 2007; Lindquist et al., 2012) view, the mental representation (valence) is specifically related to the DMN: “In our model, categorization in the form of situated conceptualization is realized in a set of brain regions that reconstitutes prior experiences for use in the present. This set of brain regions has also been called the ‘episodic memory network’ or the ‘default network’… this psychological operation makes a prediction about what caused core affective changes within one's own body or what caused the affective cues (e.g., facial actions, body postures, or vocal acoustics) in another person, and this prediction occurs in a context-sensitive way (with the result that core affect in context is categorized as an instance of anger, disgust, or fear” (Lindquist et al., 2012, p. 129). Another important network for categorization and emotional perception includes the anterior temporal lobe (ATL) and ventrolateral prefrontal cortex (VLPFC). Further, the DLPFC is postulated as being involved in mental states of attending to emotional feelings or perceptions, and holding affective information in mind in order to categorize it (Lindquist et al., 2012). Hence, the constructionist approach to emotion provides support for CSS, both regarding the categorization of emotions as being coupled with the DMN, as well as attributing to the FPN the role of mediating the activity within the two CSS spheres.

## The dynamics within the CSS

### Typical CSS dynamics

The CSS model is a dynamic system, with rich self-organizing properties. One novel aspect of CSS is the suggestion that each sphere functions as a separate dynamic system, with its own trajectory over time (Figure 4A). The two trajectories are simultaneously present, one within the inner sphere of CC/MS, and the other in the surrounding sphere of the EC/NS. These trajectories are usually antagonistic, and phenomenal awareness switches between them, as elaborated in section Second Dimension of the CSS—Awareness. In the neural space, this is manifested by the collaboration, through synchronization, of the N_i_ with either N_ms_ or N_ns_ (Figure 4B). This antagonistic behavior, however, should not be seen in early childhood.

While the detailed ontogenetic development of CSS is presently beyond the scope of this paper, we nevertheless outline in short its development, based on Heinz Werner's (1978, p. 108–109) orthogenetic principle of development, that “wherever development occurs it proceeds from a state of relative globality and lack of differentiation to a state of increasing differentiation, articulation, and hierarchic integration.” This orthogenetic principle has been shown to be consistent with the genetic organization of the cortex (Chen et al., 2012). Akin to Werner's (1978) notion of increasing differentiation and hierarchic integration, CSS is proposed to manifest with development as a successively more complex structure. In support, Anokhin et al. (2000) report that EEG dimensional complexity increases with age between 7 and 17. Moreover, the two trajectories in the CSS should, early on in development, be indistinguishable (Werner's “relative globality”), and the corresponding CSS space should comprise one global sphere (and not two). Support for this proposition was given by a recent fMRI study showing that it is only from around 2 years that the antagonistic behavior between the cortical networks is first observed (Gao et al., 2013).

There could also be states where both trajectories are under the threshold for access awareness, for example dreaming, and states where both trajectories enable access awareness, where one attends to the activity of the intrinsic system, without being immersed in it, as an observer such as in meditation. Neuroscientific studies of the neural space support this intuition: during dreaming, most of the DMN deactivates, as well as the extrinsic system (Nir and Tononi, 2010). Similarly, activity in the intrinsic system may persist in parallel to extrinsic stimulation if external stimulation is not sufficiently challenging (Greicius and Menon, 2004; Wilson et al., 2008), or when one attends to the activity of the intrinsic system (Christoff et al., 2009), as is the case during meditation (Travis and Shear, 2010). Next, we describe cases of alteration in typical CSS dynamics. All these states, we suggest, involve an alteration in the regular sense of NS, as is the case in early childhood (Oatley, 2007).

### Alterations in typical CSS dynamics

We suggest that alterations in typical CSS dynamics occur when the regular sense of NS is modified. While the typically antagonistic behavior of the trajectories essentially indicates differentiation, here we discuss those conditions wherein hierarchic integration is achieved (in Werner's terms). These states have been termed “no-self,” “transpersonal,” or “transcendent” states, and they can occur in a spontaneous or training-induced manner (Alexander and Langer, 1990; Pascual-Leone, 2000; Hartman and Zimberoff, 2008). Such states have been related to enhanced performance (Csikszentmihalyi, 1988, 1990; Leary et al., 2006) and heightened happiness (Dambrun and Ricard, 2011). We predict that these situations can be seen phenomenologically (first two predictions) as well as being translated into neural space (last two predictions), resulting in:

1. A transition toward the CC/MS trajectory being more available to access awareness;
2. An “integration” of the CC/MS and EC/NS trajectories, phenomenologically;
3. Higher activity in the neural space related to the CC/MS and lower activity in the neural space related to the EC/NS;
4. Increased synchronization between the typically antagonistic networks.

To support these propositions, we bring evidence from two distinct states, both described as involving alterations in the sense of self: optimal experience, also called flow (Csikszentmihalyi, 1988, 1990), and meditation (Hölzel et al., 2011; Fell, 2012; Vago and Silbersweig, 2012). These two states have been sometimes considered to be largely similar (e.g., Kristeller and Rikhye, 2008; Bermant et al., 2011). However, others consider flow and meditation to diverge in their phenomenology and ultimate aim, as flow fosters development through higher challenges and skill refinement while meditation mainly points toward self-transcendence (Delle Fave et al., 2011). Moreover, flow is largely spontaneous and transitory, whereas the meditative state is training-induced and can become an enduring condition. While we do not expect isomorphism between flow and meditation in the phenomenological and neural spaces, we bring both as examples of an alteration in the sense of self and increased hierarchic integration, demonstrating how we can put the CSS to work.

#### The state of flow

Flow is a state in which a person performing an activity is fully immersed in a feeling of energized focus, full involvement, and enjoyment in the process of the activity. In essence, flow is characterized by complete absorption in what one does. Conceptualized by Csikszentmihalyi (1988, 1990), flow is an optimal experience of maximum enjoyment, and a good balance between the *perceived* challenges of the task and one's own *perceived* skills. Importantly, it is a state characterized by an altered sense of self. In fact, Csikszentmihalyi (1990, p. 85) describes “excessive self-consciousness” as being the major internal obstacle to experiencing flow, as it “lacks the attentional fluidity needed to relate to activities for their own sake; too much psychic energy is wrapped up in the self, and free attention is rigidly guided by its needs.” According to Csikszentmihalyi (1988), flow is defined by eight characteristics:

1. Flow occurs when we confront challenges where we have a chance of achievement;
2. The challenges have clear goals and provide immediate feedback;
3. There is a merging of action and awareness;
4. There is intense concentration and absorption in the present moment with no intruding thoughts;
5. There is a distortion of temporal experience, one's subjective experience of time is altered, and time usually seems to pass faster;
6. The experience of the activity as autotelic—containing its own meaning and purpose, not motivated by anything beyond itself, thematically self-contained;
7. There is a loss of reflective self-consciousness;
8. There is a sense of personal control or agency over the situation or activity.

Now, we consider the arguments that support a transition toward the CC/MS trajectory being more available to access awareness (prediction 1). First, at the center of the awareness continuum, at the sharp switch from non-awareness to awareness, a person is fully immersed, concentrated and completely absorbed in an activity. This resembles the third and fourth dimensions of flow. Moreover, flow is considered to occur at a subliminal level of awareness, making the experience difficult to distinguish by recollection (Csikszentmihalyi, 1988, 1990). Similarly, Dietrich (2004) positioned flow in between maximal implicit processing and minimal explicit processing. Dietrich (2003, 2004) also related the flow experience to low DLPFC activity, calling it a state of “hypofrontality.” This is in accord with our suggestion of low N_i_ activity from the center toward the lower end of the awareness dimension.

Second, at the center of the time continuum one experiences fully the present moment. This resembles characteristics four and five, describing flow as being totally in the present moment, to the point that one experiences a distortion of time.

Third, at the center of the emotion continuum we expect to find emotional tranquility, due to a balance between negative and positive valence. This resembles characteristic number six, describing flow as being meaningful, and the general equation between flow and enjoyment. This might be a little counter-intuitive at first, as flow could be supposed to be pleasurable, hence might be placed toward the pleasant side of the emotional continuum. However, there is a distinction between pleasure and enjoyment, as emphasized by Csikszentmihalyi: “any piece of work well done is enjoyable. None of these experiences may be particularly pleasurable at the time they are taking place. Experiences that give pleasure can also give enjoyment, but the two sensations are quite different” (Csikszentmihalyi, 1990, p. 46). A recent fMRI study (Ulrich et al., 2013) investigated the neural correlates of “flow” (challenging task difficulty was dynamically adjusted to participants' individual level of skill). Comparing “flow” to “boredom” and “overload” conditions (very low and very high task demands, respectively), decreased activity was reported for the amygdala during the flow condition. Furthermore, amygdala activity was negatively correlated with subjective rating of flow. This was interpreted as indicating reduced negative arousal during the flow state, which is in accord with the CSS prediction.

Fourth, being at the center of CSS means experiencing the agentic MS, and being further away from the self-conscious NS. This is in agreement with the last two dimensions of flow, and is supported by the study of Ulrich et al. (2013), showing significantly lower mPFC activity during the flow state.

Fifth, being at the center of CSS predicts a highly embodied state. Indeed, Csikszentmihalyi (1990) describes flow states as being intimately related to the body: “It is through the body that we are related to one another and to the rest of the world. While this connection itself might be quite obvious, what we tend to forget is how enjoyable it can be. Our physical apparatus has evolved so that whenever we use its sensing devices they produce a positive sensation, and the whole organism resonates in harmony” (p. 115–116).

Up until now, we have provided evidence that the state of flow, as an example of an altered experience of the self, supports proposition 1, namely a transition toward the CC/MS trajectory being more available to the access awareness. The third characteristic of flow—“There is a merging of action and awareness,” in itself supports proposition 2 of an “integration” of the CC/MS and EC/NS trajectories, phenomenologically. As to neural space, the neuroscientific research of flow is scarce. However, the fourth proposition—namely less antagonism between the intrinsic and extrinsic networks—is given initial support by Ulrich et al. (2013), who reported three DMN regions (angular gyrus, supramarginal gyrus, and parahippocampus) to show U-shaped neural activity with increasing task difficulty, indicating lowest DMN activity during the “flow” condition, as opposed to “boredom” and “overload” conditions. Intriguingly, and counter-intuitively to the common “push-pull” antagonism notion, DMN activity is not minimal with “overload.” Taken together, the state of flow supports three of the four proposed changes in CSS and its neural space.

#### Meditation—state and trait

The word “meditation” is used to describe self-regulating practices that focus on training attention (Cahn and Polich, 2006). Meditation is expected to alter self-referential processing, as the major aim of practice is the realization, by direct experience, of the lack of any essential “self” (Dreyfus and Thompson, 2007). This has been supported by ample phenomenological studies (Austin, 2000; Leary et al., 2006; Dambrun and Ricard, 2011). Findings from meditation studies indicate training-induced neuroplasticity, both in function and in structure, (Cahn and Polich, 2006; Ivanovski and Malhi, 2007; Davidson and Lutz, 2008; Rubia, 2009).

One form of meditation that has been extensively studied is mindfulness meditation (MM), stemming from the Buddhist Theravada tradition, defined in a Western context as “the awareness that emerges through paying attention on purpose, in the present moment, and non-judgmentally to the unfolding of experience moment by moment” (Kabat-Zinn, 2003, p. 145). Looking closely at this definition, we see that it embeds training for all three CSS dimensions: awareness (awareness that emerges through paying attention on purpose), time (experience moment by moment) and emotion (non-judgmentally). We subsequently show that MM training induces a transition toward the CC/MS trajectory being more available to access awareness (prediction 1).

First, in relation to the time dimension, it was previously suggested that meditation induces a change in subjective temporal experience toward emphasizing the “now,” or being less aware of the passage of time (Brown et al., 1984). Being at the center of the time continuum in an absorbed manner can be measured as longer and longer time production (indicative of a slower rate of functioning of the internal timer, demonstrating that time seems to be moving slower; Glicksohn, 2001). In agreement with that, longer time production was shown in MM practitioners compared to control participants (Berkovich-Ohana et al., 2011), and a slower internal timer was indicated in another study (Kramer et al., 2013). Second, subjective reports on the effects of meditation have included heightened perceptual awareness (Brown, 1977; Baruss, 2003; Carter et al., 2005). This is supported by physiological studies showing MM practice to increase bodily awareness (Farb et al., 2007; Kerr et al., 2013). Third, various meditative practices were shown to lower the intensity of emotional arousal (Aftanas and Golosheykin, 2005; Nielsen and Kaszniak, 2006), to result in trait reduction in anxiety and negative affect, and an increase in positive affect (Davidson et al., 2003), and to entail lower amygdala reactivity during focused attention meditation (Brefczynski-Lewis et al., 2007). MM was also shown to increase tolerance of negative affect (Chambers et al., 2009; Farb et al., 2010), possibly by restoring balance between affective and sensory neural networks—supporting conceptual and embodied representations of emotion (Farb et al., 2012). Together, these data support prediction 1 of a transition toward the CC/MS.

In addition, accumulating evidence supports prediction 3, namely higher N_ms_ activity and lower N_ns_. For example, evidence shows that MM practice lowers the DMN (key network in N_ns_) activity (Farb et al., 2007; Pagnoni et al., 2008; Brewer et al., 2011; Berkovich-Ohana et al., 2012, 2013; Dor-Ziderman et al., 2013; summarized by Fell, 2012; Jerath et al., 2012). More specifically, various meditative techniques showed decreased activation in several areas of the DMN during practice, including the precuneus (Tang et al., 2009; Ives-Deliperi et al., 2011), mPFC (Farb et al., 2007; Brewer et al., 2011; Ives-Deliperi et al., 2011), PCC (Pagnoni et al., 2008; Tang et al., 2009; Brewer et al., 2011), ACC (Pagnoni et al., 2008; Ives-Deliperi et al., 2011;) and LTC (Pagnoni et al., 2008). A similar result was shown using EEG, where lower frontal-midline gamma power (Berkovich-Ohana et al., 2012), or lower gamma functional connectivity (Berkovich-Ohana et al., 2013) were indicative of lower trait DMN activity.

Finally, several recent fMRI studies support prediction 4, namely increased synchronization between the typically antagonistic networks. An fMRI study showed a stronger coupling between the intrinsic and extrinsic systems during non-dual meditation (Josipovic et al., 2011). Brewer et al. (2011) reported that the correlation between areas involved in cognitive control (dACC, DLPFC), which are part of N_i_, and the PCC area in the N_ns_ were higher for experienced meditators than controls, both at rest and during meditation. Additionally, it was shown that during MM practice, as compared to rest, functional connectivity is strengthened between the DAN (comprising N_ms_) and DMN (N_ns_) (Froeliger et al., 2012). To summarize this section, we have provided support from meditation research for three predictions.

## A comparison to other models of consciousness

This section posits CSS in the wider context of different approaches to consciousness. Obviously, other theories are mentioned here briefly, as their elaboration is beyond the scope of this paper. CSS provides a phenomenological map which includes all possible consciousness states. It suggests that all possible states of consciousness are a combination of three dimensions, and that each consciousness state involves a specific sense of selfhood. It also describes the system's dynamic behavior. Furthermore, it provides a tentative neural space. Such a description is totally missing in the literature. However, it builds on previous theories of consciousness, which describe some of these dimensions at a time, as outlined below.

Various theories of consciousness suggesting functional descriptions have emerged in the last decade (reviewed by Lau and Rosenthal, 2011; and by Seth et al., 2008). These include neurodynamical approaches to consciousness (Varela et al., 1991, 2001; Tononi and Edelman, 1998; Dehaene and Naccache, 2001; Dehaene and Changeux, 2005; Tononi, 2008). Despite their differences, these various models agree that the constitution of dynamic spatiotemporal patterns of neural activity, namely neuronal synchrony, plays a central role in the emergence of consciousness (reviewed by Cosmelli et al., 2007). Specifically, these theories explain what differentiates conscious experience from subliminal or un-conscious experience (Dehaene et al., 2006), why thalamocortical anatomy suits conscious experience as opposed to different neural architectures such as seen in the cerebellum, or afferent and efferent pathways (Tononi, 2008), and how the proposed gap between qualia and brain activity (Chalmers, 1995) can be reduced. CSS builds on Tononi's (2008) integrated information theory, accepting that the neural space is mainly attributed to thalamocortical loops, that consciousness arises from integrated informational relationships generated by a complex of elements in the neural space (Tononi's “main complex”), and that the larger the complex, the greater the information the system can generate. CSS sees consciousness as essentially embodied, as previously emphasized by others (Varela et al., 1991; Thompson and Varela, 2001; Cosmelli and Thompson, 2010). CSS also incorporates workspace theory (Dehaene et al., 2006), as elaborated in the section on awareness. However, the CSS model departs from these theories, by proposing to explain phenomenologically all possible states of consciousness, as well as suggesting a possible neural space.

Other phenomenological accounts describe states of consciousness along a continuum of experience of the self. Some theories emphasize the awareness dimension, including those presented by Schooler (2002), Brown (1976), and Natsoulas (1998), which are then integrated into a unifying social/personality model describing degrees of consciousness and selfhood (Morin, 2006). Other theories emphasized the importance of time (including Neisser, 1988; Stuss et al., 2001; Newen and Vogeley, 2003; Zelazo, 2004) or emotions (including Lambie and Marcel, 2002; Panksepp, 2005; Barrett et al., 2007; Tsuchiya and Adolphs, 2007) in relation to selfhood. In relation to these models, CSS extends the experience of selfhood to encompass both the time and emotion dimensions in one coherent framework.

Importantly, Damasio (1999, 2012) provides a neuroscientific account for consciousness and self, which has inspired our model, although we departed from it substantially, as subsequently elaborated. Damasio outlines and combines two theories (Dolan, 1999); the first concerns the propagation of consciousness and self from body along a continuum: from (1) an unconscious bodily self (proto-self), which deals with the state of the internal milieu and creates a first-order representation of current body states in the brain, to (2) the core-self (CS), which gives rise to core-consciousness (CC). CC is a complex of second-order mental maps based in the feeling state, which arises when the proto-self interacts with the first-order sensory maps that represent objects; and finally, (3) Extended consciousness (EC), which depends on CC, deals with holding in mind, over time, a multiplicity of neural patterns that describe the autobiographical-self (AS). AS is heavily dependent on the formation of enduring experiential memories, attention and language, and its inevitable concomitant is personal identity. Damasio's second theory concerns affect, and has been referred to briefly before (section Third Dimension of the CSS—Emotion). To enable a careful comparison of CSS with Damasio's view, we refer the reader to Table 1.

**Table 1:** **A comparison between Damasio's (1999) theory of consciousness and the CSS model, describing points of departure**.

**Dimension**	**Damasio's view**	**CSS model—points of departure**
CC	Stable across the lifetime of the organism; it is not exclusively human; and it is not dependent on conventional memory, working memory, reasoning, or language	Phenomenological space can increase with mental training, dependent on working memory and when involves awareness, dependent on attention
3 types of self	Proto, unconscious	Body, unconscious
	Core, involves CC, conscious	Minimal, can be either aware or unaware
	Autobiographic, involves EC, conscious	Narrative, can be either aware or unaware
Types of affect	Emotions—bodily, public, primary or secondary emotions, can be non-conscious, involves CS	Arousal—involves core affect, can be either aware or unaware
	Feeling—private, mental experiences of an emotion, can be non-conscious, on the boundary between CS and AS	Valence—involves conceptualization, can be either aware or unaware
	Feeling of feeling—involves AS and conscious experience	

*CC, core consciousness; EC, extended consciousness; CS, core self; AS, autobiographic self*.

Damasio's view is largely adopted in CSS concerning the CC and EC, and their respective type of selfhood. A major difference is positioning both types of consciousness and self on an awareness continuum, where both can be either conscious or non-conscious, as opposed to the gradual propagation of conscious experience described by Damasio. A second major departure from Damasio's view concerns emotions, his three levels of emotion being replaced by the two dimensions of valence and arousal. As a result of these differences, when the linear view of Damasio is replaced by the 3D view of the CSS, more flexibility is available to the system, and explanatory power increases. For example, Damasio's view could not explain unaware emotional mental evaluation (Winkielman and Berridge, 2004; Sato and Aoki, 2006), unaware mental representation of numbers (Greenwald et al., 2003; Ric and Muller, 2012) or unaware semantic priming (Dehaene et al., 1998; Naccache and Dehaene, 2001).

To conclude this section, the relationship of CSS to other models of consciousness has been elaborated, especially with regard to Damasio's view. This comparison was intended to highlight the integrative, unifying and explanatory power of the CSS model.

## The limitations, predictions and contribution of the CSS model

The CSS model has several limitations, which warrant delineation. First, CSS describes a phenomenological space, and also attempts to suggest its neural space. The neural space described here should be regarded as a coarse attempt based on current understanding. Second, the resolution between states in phenomenal space is much higher than current resolution in neural space (as already discussed by Fell, 2004), rendering a full translation from the phenomenological to the neural space impossible at the moment. Third, the important issue of how core cognitive functions, such as emotion regulation and reward, are created by the interplay between the three dimensions was left out. Finally, the presentation of the CSS model in this article leaves some important issues untouched, including: (1) Individual differences in the size and shape of CSS; (2) Pathologies of consciousness and selfhood; (3) Elaboration on the neural reference space to include electrophysiological activity; and (4) The location of altered states of consciousness within the CSS, an issue strongly related to the “breakdown” of the time dimension. These issues would hopefully be the topic of further developments of the model.

We conclude by providing examples of several predictions of the model which relate to current debates in the literature, and which are scientifically testable. First, given that an experience can fall into any coordinate in CSS, the model predicts some experiences which are still being debated. For example, albeit the controversy about the very existence of unconscious emotions (e.g., Clore, 1994; Winkielman and Berridge, 2004), the model predicts that full-blown emotions (including hedonic feeling and appraisal) could be experienced without access awareness. Second, based on the dual phenomenological composition of each of the three dimensions, CSS predicts that their interaction in experience (access awareness) takes place only in the same sphere. For example, during emotion evaluation (outer sphere), one cannot simultaneously process external output (inner sphere). Or if one experiences core emotions/arousal (inner sphere), one cannot experience simultaneously mental time traveling (outer sphere). Obviously, this bears consequences for the neural spaces' interaction, requiring antagonism between the corresponding structures (as described in this paper), which could be readily addressed empirically by blood oxygenated level dependent (BOLD) fMRI studies. Third, CSS predicts that any well-reasoned condition in which one would expect hierarchic integration and alteration in the regular sense of NS, such as flow and meditation, will exhibit the four predictions laid out in section Typical CSS Dynamics, including that the two neural spaces should be positively correlated. Fourth, a prediction concerning complexity can be derived from CSS development (section The Dynamics within the CSS). As CSS manifests with development as a successively more complex structure, it predicts not only increasing complexity with age, but decreasing complexity in conditions of hierarchic integration, such as flow and meditation (for a similar view, see Sharp, 2011). For example, children should have a CSS which is more “global” in a Wernerian sense (hence, less complex), and meditators should have a CSS which is more hierarchically integrated (hence, also less complex). Partial support for this prediction was given by a finding of lower dimensional complexity during meditation, compared to rest (Aftanas and Golocheikine, 2002).

The model presented here creates a broad theoretical framework with explanatory and unificatory power, that attempts to make sense of a wide range of otherwise unrelated phenomenological and neuroscientific observations. Importantly, the model provides a new framework for understanding the relationship between core aspects of consciousness, hence lays a theoretical basis for the study of consciousness. We hope this model will inform future studies, and raise further testable predictions.

## Author contributions

Aviva Berkovich-Ohana conceptualized the model and wrote the paper. Joseph Glicksohn contributed to the conception of the work and revised all its versions critically.

### Conflict of interest statement

The authors declare that the research was conducted in the absence of any commercial or financial relationships that could be construed as a potential conflict of interest.
